# An Inflammatory Nucleus Pulposus Tissue Culture Model to Test Molecular Regenerative Therapies: Validation with Epigallocatechin 3-Gallate

**DOI:** 10.3390/ijms17101640

**Published:** 2016-09-27

**Authors:** Olga Krupkova, Marian Hlavna, Julie Amir Tahmasseb, Joel Zvick, Dominik Kunz, Keita Ito, Stephen J. Ferguson, Karin Wuertz-Kozak

**Affiliations:** 1 Institute for Biomechanics, ETH Zurich, Hoenggerbergring 64, CH-8093 Zurich, Switzerland; hlavna.marian@gmail.com (M.H.); ajulie@student.ethz.ch (J.A.T.); jzvick@student.ethz.ch (J.Z.); kunzdo02@students.zhaw.ch (D.K.); sferguson@ethz.ch (S.J.F.); kwuertz@ethz.ch (K.W.-K.); 2 Health Department, ZHAW—Zurich University of Applied Sciences, Technikumstrasse 71, CH-8401 Winterthur, Switzerland; 3 Department of Biomedical Engineering, Eindhoven University of Technology, Postbus 513, 5600 MB Eindhoven, The Netherlands; K.Ito@tue.nl; 4 Competence Center for Applied Biotechnology and Molecular Medicine, University of Zurich, Winterthurerstrasse 190, CH-8057 Zurich, Switzerland

**Keywords:** nucleus pulposus, organ culture model, degenerative disc disease, IL-1β, TNF-α, chondroitinase ABC, epigallocatechin 3-gallate, inflammation, extracellular matrix

## Abstract

Organ cultures are practical tools to investigate regenerative strategies for the intervertebral disc. However, most existing organ culture systems induce severe tissue degradation with only limited representation of the in vivo processes. The objective of this study was to develop a space- and cost-efficient tissue culture model, which represents degenerative processes of the nucleus pulposus (NP). Intact bovine NPs were cultured in a previously developed system using Dyneema jackets. Degenerative changes in the NP tissue were induced either by the direct injection of chondroitinase ABC (1–20 U/mL) or by the diffusion of interleukin-1 beta (IL-1β) and tumor necrosis factor alpha (TNF-α) (both 100 ng/mL) from the culture media. Extracellular matrix composition (collagens, proteoglycans, water, and DNA) and the expression of inflammatory and catabolic genes were analyzed. The anti-inflammatory and anti-catabolic compound epigallocatechin 3-gallate (EGCG, 10 µM) was employed to assess the relevance of the degenerative NP model. Although a single injection of chondroitinase ABC reduced the proteoglycan content in the NPs, it did not activate cellular responses. On the other hand, IL-1β and TNF-α significantly increased the mRNA expression of inflammatory mediators IL-6, IL-8, inducible nitric oxide synthase (iNOS), prostaglandin-endoperoxide synthase 2 (PTGS2) and matrix metalloproteinases (MMP1, MMP3, and MMP13). The cytokine-induced gene expression in the NPs was ameliorated with EGCG. This study provides a proof of concept that inflammatory NP cultures, with appropriate containment, can be useful for the discovery and evaluation of molecular therapeutic strategies against early degenerative disc disease.

## 1. Introduction

The intervertebral disc (IVD) consists of three structurally and functionally different tissues: the outer annulus fibrosus (AF), the inner nucleus pulposus (NP), and two cartilaginous endplates that connect the disc with the adjacent vertebrae [1,2]. IVD degeneration is caused by a combination of many events throughout a lifetime, including non-physiological loading, endplate calcification, immune reactions, cell senescence, and cell death [1,3,4]. These events reduce the repair capacity of the IVD, induce inflammation and cause a catabolic shift in the NP tissue.

The inflammatory cytokines interleukin-1 beta (IL-1β) [5,6] and tumor necrosis factor alpha (TNF-α) [7] are key molecules involved in IVD degeneration. The events that promote their production by NP and AF cells, especially in the absence of trauma or herniation, can be chronic stress, spinal infection or swelling [3,8,9]. IL-1β and TNF-α bind to their cell surface receptors (e.g., IL-1R1, and TNFR1), which activate the signaling mediators nuclear factor κB (NF-κB), C-jun-N-terminal kinase (JNK), and p38, resulting in the transcription of inflammatory and catabolic genes such as interleukins (IL-1α, IL-1β, IL-6, IL-8), matrix metalloproteinases (MMP1, MMP3 and MMP13), aggrecanases (a disintegrin and metalloproteinases with thrombospondin motifs, ADAMTS) and other relevant molecules that can promote inflammation and pain [inducible nitric oxid synthase (iNOS), prostaglandin-endoperoxide synthase 2 (PTGS2) and nerve growth factor (NGF)] [3,10,11,12]. In addition to the catabolic and pro-inflammatory effects, IL-1β and TNF-α can influence disc cell senescence, autophagy and the expression of genes involved in proliferation [13]. MMPs and aggrecanases further promote degradation of the extracellular matrix (ECM) [10,14,15]. Changes of the NP ultimately result in a loss of disc hydration, load-bearing capacity, and disc height at later stages [16,17]. These accelerated pathological processes with structural failure and the development of lower back pain, e.g., due to nerve compression and/or chemical irritation, are together termed degenerative disc disease, a distinct entity to the process of physiological ageing [16,18,19,20].

Despite an increasing prevalence of discogenic back pain and the consequently high economic burden [21], commonly used therapies do not target the causes of degenerative disc disease. Both conservative and surgical treatments are mainly focused on the resolution of pain [22,23]. Current preclinical research explores new strategies for restoring the function and homeostasis of degenerated discs via inhibition of inflammation, prevention of premature ageing, and augmentation of the ECM content. Cell therapies (e.g., mesenchymal stem cells) can support the existing cell population and “rejuvenate” the aging disc [24,25]. However, newly implanted cells are at risk of undergoing apoptosis, due to the surrounding inflammatory and catabolic environment [26]. Inflammation, catabolism and matrix degradation in the disc can be controlled using molecular therapeutics, such as anti-inflammatory compounds [11,27], growth factors [28,29] and N-terminus of Link protein (LinkN) [30]. Application of growth factors like bone morphogenetic proteins (BMP-2, and BMP-7), insulin growth factor (IGF-1) or tumor growth factor (TGF-β3) can induce the formation of new ECM and improve the mechanical properties of the disc [31]. However, drawbacks of exogenous growth factors are their short half-life (hours to days) and possibly only transient effects. Another complication is their large size, which can limit their diffusion through the NP [32].

Diverse signaling pathways are deregulated in disc degeneration. Therefore, small molecules with multiple effects, targeting both survival and function of resident disc cells, are promising therapeutic candidates. Compounds derived from traditional medicinal plants act on several signal transduction and apoptotic cascades at the same time, while having minimal side effects [33]. We have shown before that natural compounds such as resveratrol [34], curcumin [35], triptolide [36] and epigallocatechin 3-gallate (EGCG) [11,37] exhibit anti-inflammatory, anti-catabolic and anti-oxidant activity in disc cells. Although more research is needed to fully understand the effects of these compounds on the integrity of the IVD, it has been suggested that they can delay degeneration of the NP or even reverse it. Natural small molecules can also enhance disc nutrition via improving vascular health [38] and relieve radicular pain [11,34].

A number of organ culture models have been developed over the past years to facilitate testing of novel regenerative treatments. Unlike cell cultures, organ culture models retain the native ECM and allow for mechanical loading [39]. Induction of degeneration in these models can be achieved by injection of ECM degrading enzymes such as trypsin [40,41] and papain [42,43]. Enzymatic tissue models allow efficacy evaluation of cell therapies. Cells injected or implanted within a supportive scaffold can fill in the missing ECM, proliferate, produce new matrix and restore tissue properties. However, while ECM degradation is often extensive, and thus higher than clinically observed, no or limited cellular inflammatory and catabolic reactions have been found in these models. This suggests that in vivo disc degeneration is not fully mimicked, making enzymatic models less efficient for testing molecular therapeutics. Mechanical damage such as endplate fracture [44] or stab incision in animal models [45] can activate cellular responses in the disc more reliably than non-physiological proteolytic enzymes. Recently, a stab injury model, based on motion segments isolated from transgenic animals with an NF-κB-luc reporter, was developed. Longitudinal monitoring of NF-κB activity in this model can provide insights into the mechanisms that regulate IVD inflammatory responses to stab injury [46]. At the same time, physiologically relevant cytokine-induced inflammatory organ culture models, without involvement of mechanical damage, have emerged [7,47,48].

Whole disc organ cultures, derived from large animals and containing biologically relevant ECM degradation and inflammation, are certainly most suitable for testing regenerative strategies for the disc. However, although whole disc organ cultures have many advantages, they typically do not allow for high throughput experiments due to space-related demands and sample heterogeneity. To overcome this issue, a nucleus pulposus (NP) culture in Dyneema jackets has been recently developed [49], (further referred to as “NP culture”). The Dyneema jackets (also called “artificial AF”) restrain swelling, while maintaining diffusion of nutrients from culture media. This bovine NP culture was previously shown to preserve a glycosaminoglycan (GAG) content and gene expression pattern similar to native tissue over sustained periods [49]. The objective of this work was to induce degenerative changes in the existing NP culture, establishing a platform for high-throughput tests of regenerative treatments.

We hypothesized that: (1) physiological degradation of the ECM can be induced by injection of the biologically relevant enzyme chondroitinase ABC; (2) inflammatory and degenerative responses can be activated by diffusion of IL-1β and TNF-α from culture media; and (3) the anti-inflammatory and anti-catabolic compound EGCG will ameliorate this degenerative response. Using bovine caudal NPs for preclinical testing is not only cost-effective, but also in accordance with the 3R (Replace, Reduce, Refine) animal welfare recommendations.

## 2. Results

### 2.1. Nucleus Pulposus Tissue in Dyneema Jackets Is Stable over a Period of 21 Days

**Figure 1:**
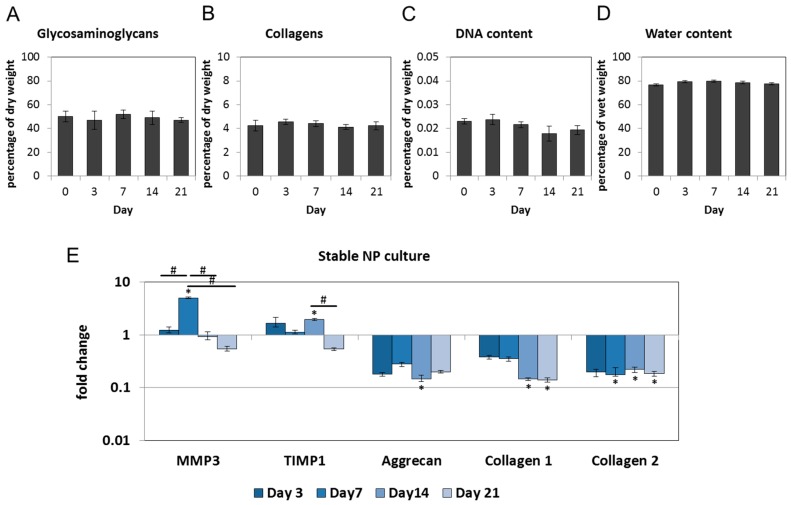
Bovine nucleus pulposus (NP) culture preserves extracellular matrix over a period of 21 days: (**A**) Glycosaminoglycan; (**B**) collagen; (**C**) DNA; and (**D**) water content on Days 3, 7, 14 and 21 did not differ from the native values (Day 0). Graphs show the percentage of dry or wet weight relative to the native tissue (mean ± SEM, *n* = 4 per group); (**E**) gene expression of matrix metalloproteinase 3 (MMP3) and tissue inhibitor of matrix metalloproteinases (TIMP1) increased and subsequently returned close to the native levels. Gene expression of aggrecan, collagen 1 and collagen 2 was down-regulated after NPs were placed in culture and remained stable thereafter. Graph shows fold change relative to the native tissue (mean ± SEM, *n* = 4–8 per group). * *p* < 0.05 vs. native tissue; ^#^ *p* < 0.05 vs. each other (ANOVA).

GAG, collagen, DNA and water were determined in the NP cultures on Days 3, 7, 14 and 21 and compared with native tissue (Day 0). The content of GAG, collagen, DNA and water in the NP cultures did not significantly differ from the native tissue, confirming stability of the ECM [49] (Figure 1A–D). Gene expression of MMP3 and tissue inhibitor of matrix metalloproteinases (TIMP1) increased on Days 3 and 7, which may reflect a tissue response to culture conditions. Their expression was downregulated at later time points (Figure 1E). Genes for other matrix degrading enzymes (MMP1, and MMP13) and interleukins (IL-6, IL-8, and IL-1β) were not detectable. In comparison with the native tissue, gene expression of aggrecan and collagens decreased between Day 0 and 3, which can be explained by the absence of physiological loading [49]. After this initial down-regulation, the expression of ECM genes remained stable (Figure 1E). Average cycle threshold values (Cq ± SD) are listed in Table 1.

### 2.2. Chondroitinase ABC Reduces Proteoglycan Content in the NP but Does Not Induce Cell Responses

**Figure 2:**
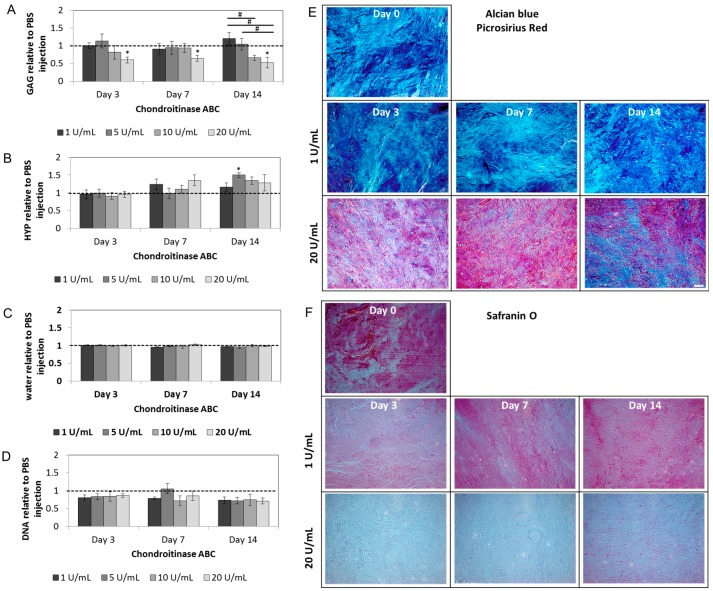
Chondroitinase ABC reduces proteoglycan content in the NP: (**A**) as expected, the glycosaminoglycan content significantly decreased in the NPs injected with 20 U/mL Chondroitinase ABC compared with the NPs injected with PBS (set as 1); (**B**) collagens; (**C**) DNA content; and (**D**) water content remained unchanged. Graphs show ECM composition relative to the PBS injection (mean ± SEM, *n* = 4 per group); (**E**) Alcian blue/Picrosirius red; and (**F**) Safranin O staining confirmed that 1 U/mL chondroitinase ABC did not have major proteoglycan-degrading effects, whereas 20 U/mL reduced glycosaminoglycan (GAG) content at all tested time points. Scale bar, 100 µm. * *p* < 0.05 vs. PBS injection (dashed lines); ^#^ *p* < 0.05 vs. each other (ANOVA).

**Figure 3:**
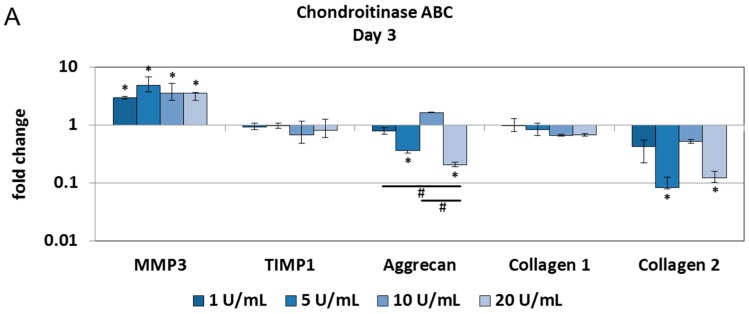

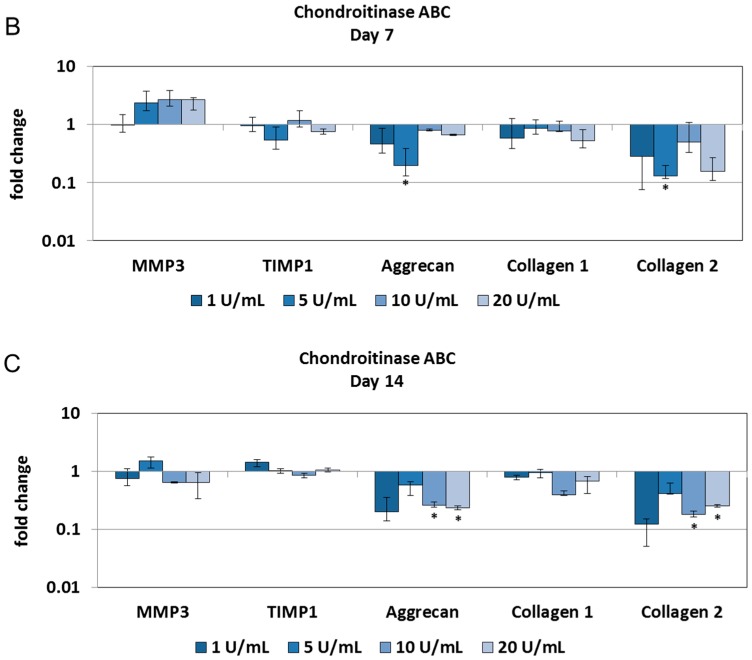
Chondroitinase ABC does not activate cell reactions in the NP: Injection of 1–20 U/mL chondroitinase ABC reduced gene expression of aggrecan and collagen 2 in the NP cultures on Days 3 (**A**); 7 (**B**); and 14 (**C**). Gene expression of MMP3 increased on Day 3, but was downregulated at later time points. Gene expression of TIMP1 and collagen 1 remained close to the control levels. Inflammatory and catabolic markers were not detectable. Graphs show fold change relative to the PBS injection (mean ± SEM, *n* = 4). * *p* < 0.05 vs. PBS injection; ^#^ *p* < 0.05 vs. each other (ANOVA).

Physiological degradation of ECM was induced by chondroitinase ABC. As chondroitinase ABC removes chondroitin sulfate from proteoglycans, a dose- and/or time-dependent decrease in sulfated GAGs was expected. Compared with the phosphate buffered saline (PBS) injection, the injection of 20 U/mL chondroitinase ABC significantly reduced GAG content at all tested time points (Figure 2A). No major alterations in collagen, DNA and water content were detected over a period of 14 days (Figure 2B–D). Alcian blue/Picrosirius red and Safranin O staining confirmed that 20 U/mL of chondroitinase ABC depleted proteoglycans, whereas 1 U/mL chondroitinase ABC retained a macromolecular composition closer to the native tissue (Figure 2E,F). However, gene expression analysis revealed no profound changes in the expression of inflammatory and catabolic genes. Genes for matrix degrading enzymes (MMP1, and MMP13) and interleukins (IL-6, IL-8, and IL-1β) were not detectable. Chondroitinase ABC downregulated the expression of collagen 2 and aggrecan, but the expression of collagen 1 remained unchanged (Figure 3A–C).

### 2.3. Interleukin-1 Beta (IL-1β) and Tumor Necrosis Factor Alpha (TNF-α) Induce Gene Expression of Inflammatory and Pain-Related Markers in the NP Cultures

**Figure 4:**
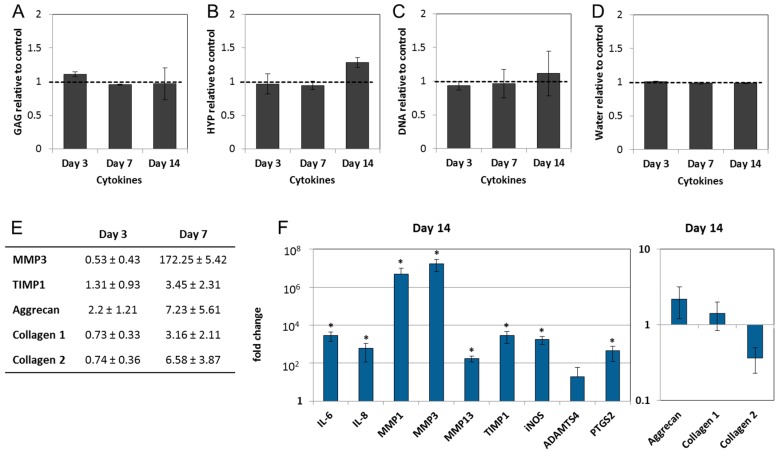
IL-1β and TNF-α induce an inflammatory and catabolic phenotype in the NPs: (**A**) Glycosaminoglycan; (**B**) collagen; (**C**) DNA; and (**D**) water content in cytokine-treated NPs remained unchanged over a period of 14 days, when compared with the untreated group (set as 1). Graphs show ECM composition relative to untreated samples (mean ± SEM from *n* = 4); (**E**) inflammatory and catabolic gene expression was not detectable on Days 3 and 7, and the expression of MMP3, TIMP1 and ECM macromolecules were close to the control values (fold change); (**F**) on Day 14, IL-1β and TNF-α significantly induced gene expression of interleukins (IL-6, IL-8), collagenases (MMP1, MMP3, MMP13) and their tissue inhibitor (TIMP1), aggrecanase 1 (ADAMTS4) and pain mediators (PTGS2, iNOS). Graphs show fold change relative to the untreated samples (mean ± SEM from *n* = 4–7). * *p* < 0.05 vs. untreated control (ANOVA).

Cytokines IL-1β and TNF-α are known activators of inflammatory and catabolic cascades in the IVD and their expression may affect ECM composition at later stages. However, during 14 days of culture, IL-1β and TNF-α did not influence GAG, collagen, DNA and water content in the NPs (Figure 4A–D). Gene expression analysis revealed that on Days 3 and 7 cytokine-treated NPs expressed low levels of MMP3, TIMP1 and ECM macromolecules, similarly to the stable and enzyme-treated NP cultures (Figure 4E). On the other hand, cytokines significantly induced the expression of pro-inflammatory interleukins (IL-6, and IL-8), collagenases (MMP1, MMP3, and MMP13), their inhibitor TIMP1, and pain-related mediators (iNOS, and PTGS2) on Day 14 (Figure 4F). These results indicate that a catabolic/inflammatory shift towards a degenerative phenotype developed between Day 7 and 14. Changes in the expression of the ECM macromolecules may suggest remodeling attempts in response to the inflammatory stimuli. Average Cq ± SD values for all genes in all treatment groups are shown in Supplementary Table S1.

### 2.4. Epigallocatechin 3-Gallate Ameliorates the Inflammatory and Catabolic Phenotype in the NP Cultures

**Figure 5:**
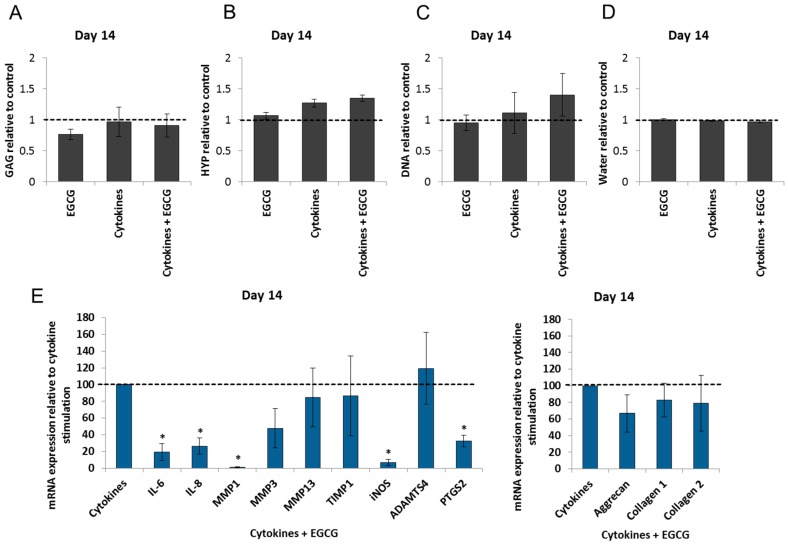
Epigallocatechin 3-gallate ameliorates inflammatory and catabolic responses in the NPs: (**A**) Glycosaminoglycan; (**B**) collagen; (**C**) DNA; and (**D**) water content in the NPs treated with EGCG, IL-1β and TNF-α, and their combination remained unchanged over a period of 14 days. Graphs show ECM composition relative to the untreated samples (mean ± SEM from *n* = 4); (**E**) on Day 14, EGCG reduced the expression of interleukins (IL-6, IL-8), collagenases (MMP1, MMP3) and pain mediators (PTGS2, iNOS) in cytokine-treated NPs. Gene expression of ECM components remained close to the control values. Graphs show mRNA expression relative to the cytokine-stimulated samples (mean ± SEM from *n* = 4–7). * *p* < 0.05 vs. IL-1β and TNF-α treatment (*t*-test).

As a significant up-regulation of inflammatory and catabolic genes was detected in the IL-1β and TNF-α-treated NP cultures on Day 14, this experimental group was subjected to anti-inflammatory treatment. Culture media was supplemented with EGCG (10 µM) on Day 0 and 7. Neither EGCG alone nor in combination with IL-1β and TNF-α significantly influenced the composition of the ECM (Figure 5A–D) or the gene expression of ECM macromolecules. EGCG inhibited the expression of inflammatory markers (IL-6, and IL-8), catabolic enzymes (MMP1, and MMP3), and pain mediators (iNOS, and PTGS2). This not only provided evidence on the activity of EGCG in the NP tissue, but also proved the suitability of the inflammatory NP cultures for evaluating the effects of potential molecular therapeutics (Figure 5E).

## 3. Discussion

Throughout a lifetime, lumbar intervertebral discs undergo a morphological and functional decline. Early signs of disc degeneration, caused by impaired exchange of solutes and catabolic shift, occur in the NP [50]. In some individuals, NP degeneration leads to internal disc disruption and annulus fibrosus damage, which can result in lower back pain. Although discogenic back pain constitutes a rising socioeconomic burden for Western countries, therapies targeting its biological causes are lacking. The aim of this study was to develop an organ culture model, which will allow preclinical screening of novel molecular therapeutics for the degenerative nucleus pulposus.

A previously validated system that employs bovine caudal NPs cultured in Dyneema jackets (“artificial AF”) was used [49]. Although bovine caudal discs are not weight bearing, they have been proposed as a suitable biological model for human disc degeneration, due to their close size, comparable cellularity and similar in vivo pressure [51]. The bovine NP culture potentially represents a space-efficient and relatively inexpensive approach to screen for molecular therapeutics that can be used in human medicine. Stable bovine and human NP cultures were used for investigating the effects of ECM anabolics such as LinkN, notochordal cell-conditioned medium [52] and bone morphogenic protein 7 (BMP-7) [53]. However, the stable bovine NP culture exhibits neither an inflammatory phenotype nor ECM degradation. Inducing biologically relevant degenerative changes in this model will provide a platform for testing compounds that not only activate ECM production, but also interfere with inflammation, catabolism and pain on the cellular level.

Although validated, NP cultures may not completely preserve the native tissue characteristics, as a decrease in GAG content and an increase in gene expression of MMPs have been described, especially at later time points [49]. Therefore, our initial experiments aimed to test NP culture stability over a period of 21 days, to evaluate its relevance for development towards a degenerative phenotype. In accordance with the previously published study, we observed an initial downregulation of ECM genes and a transient increase in the expression of MMP3, likely caused by a lack of mechanical loading. As stabilization of these parameters followed, the NP culture was considered suitable for optimization towards a degenerative model.

ECM degrading enzymes that are commonly used to induce proteoglycan depletion in the NP are the protein hydrolases trypsin and papain. However, as there is no naturally occurring proteolysis in the disc, these enzymes do not activate cell reactions such as the expression of interleukins and MMPs, but induce excessive matrix degradation, with a low physiological fidelity. On the other hand, enzymes that degrade extracellular matrix in a biologically relevant manner can possibly induce cellular responses that are found during disc degeneration. Recently MMP3, ADAMTS4, and HTRA1 were injected into bovine discs and cultured for eight days. Even though these enzymes did not affect matrix degradation and gene expression, they reduced cellular metabolic activity. HTRA1 additionally caused a loss of disc height and was shown to induce MMP expression in another in vitro study [54,55]. In our study, chondroitinase ABC with lyase activity was employed to facilitate GAG depletion. Chondroitinase ABC degrades polysaccharides, reproducing age-related removal of chondroitin sulfate from aggrecan [56]. However, although injection of chondroitinase ABC accelerated degradation of GAGs in a biologically relevant manner, it was not sufficient for activation of inflammation and catabolism in the NP tissue. This confirms previous observations that injection of the ECM degrading enzyme alone does not entirely mimic the degenerative process. Therefore, chondroitinase ABC-injection into a NP culture is not entirely suitable when the aim is to create a tissue culture system for testing bio-active compounds.

The involvement of pro-inflammatory cytokines in painful degenerative disc disease is well established [3]. TNF-α and IL-1β can act as nociceptive triggers as well as induce the expression of other potentially nociceptive interleukins (such as IL-6, IL-8, IL-12, and IL-17) and direct pain mediators (nitric oxide, prostaglandin E, and substance P) [13,57,58,59,60,61]. Degenerated discs with newly formed nociceptive nerve fibers can thus be a primary source of back pain [62]. Moreover, disc degeneration also can result in compression of nerve roots and spinal nerves. IL-1β, TNF-α and other pain-mediating chemicals released from the NP can irritate dorsal root ganglions, contributing to functional and structural nerve damage, radiculopathy and muscle weakness in vivo [57,63,64]. Replicating such an inflammatory phenotype in organ cultures is therefore needed for a successful drug discovery process.

In the study of Purmessur et al. [7], TNF-α (200 ng/mL) activated the expression of inflammatory and catabolic genes and accelerated degradation of aggrecan in bovine explants. Similarly, in another study, IL-1β up-regulated pro-inflammatory markers (IL-6, IL-8, and prostaglandin E2) and MMPs in punctured bovine discs, compared with disc organ cultures treated with needle puncture alone [48]. In our study, a combination of IL-1β and TNF-α (100 ng/mL each) strongly induced the expression of interleukins (IL-6, and IL-8), collagenases (MMP1, MMP3, and MMP13), aggrecanase 1 (ADAMTS4) and molecules associated with pain sensation (iNOS, and PTGS2). The inflammatory and catabolic phenotype in our NP cultures fully developed on Day 14, possibly due to the dense proteoglycan network in initially healthy bovine NP tissue, as proteoglycans in healthy NPs can slow down cytokine penetration [32]. In smaller rat lumbar discs (±0.5 cm in diameter), TNF-α and IL-1β activated the expression of MMPs and ECM changes already after three days [65], most probably due to shorter diffusion distances [51]. Mechanical loading of the bovine NP cultures in future studies may aid to promote cytokine penetration and activate responses at earlier time points.

IL-1β and TNF-α-induced inflammation did not affect composition of the ECM over 14 days. This can be explained by limited MMP activity as well as overall low expression of MMPs in the NP tissue. Although the fold change between control group and inflammation group was high, the absolute amount of MMPs can still be low, resulting in unchanged ECM structure. Another reason can be elevated expression of TIMP1. It has been shown before that healthy disc cells respond to stress by upregulation of matrix metalloproteinase inhibitors (TIMPs) [66], which inhibit the activity of MMPs in a 1:1 stoichiometry. In particular, TIMP-1 forms a complex with the catalytic domain of MMP-3, reducing its activity in the NP tissue [54]. Therefore, the inflammation-related loss of ECM in our model is expected to occur at later time points. Although ECM degradation has not been observed in the cytokine-treated group, the current inflammatory bovine NP model can be used to screen therapeutics against early stages of disc degeneration, before the loss of ECM occurs. Another advantage of our bovine NP culture model is that molecular diffusion distances in bovine NPs are relevant to human NPs [51]. An interesting option would be to combine chondroitinase ABC injection with cytokine treatment. Biologically relevant proteoglycan depletion, accompanied by inflammatory and catabolic cell reactions, could accurately reproduce more advanced stages of NP degeneration. Possibilities to replicate different stages of degeneration in the NP cultures are currently being investigated in our group.

In the past, we have identified the polyphenol epigallocatechin gallate (EGCG) as a promising compound for the treatment of painful degenerative disc disease. EGCG inhibited the expression of inflammatory markers (IL-6, IL-8, PTGS2, TLR2, and iNOS), collagenases (MMP1, MMP3, and MMP13) and NGF in human IVD cells cultured adherently and in alginate beads [11]. EGCG also protected IVD cells from oxidative stress caused by exogenous radicals [37] and, moreover, locally delivered EGCG reduced radicular pain in a rat model of NP herniation [11]. Therefore, we selected this compound for validation of the new inflammation-induced NP culture model. EGCG treatment of NP cultures exposed to IL-1β and TNF-α reduced the expression of interleukins, MMPs, iNOS and PTGS2, which confirmed our results from previous in vitro tests. In addition to in vitro studies, the NP culture system revealed no negative effects of EGCG on the extracellular matrix, further supporting future research on this compound. As EGCG ameliorated the expression of inflammatory markers in the tissue, the NP culture system proved suitable for preclinical testing of bioactive compounds and possibly also compound combinations and slow release systems.

Similar effects of EGCG have been shown before in cell culture studies. However, human NP contains only less than 1% cells [17]. Therefore, organ culture study should be performed in order to assess the possible effects of EGCG on the ECM. This provides information on the overall efficacy and clinical relevance of EGCG, before designing animal tests. To our knowledge, this is the first study presenting the effects of EGCG on inflammatory intervertebral disc tissue.

In comparison with animal models, organ culture systems represent a cost-effective alternative for discovery and testing of novel therapeutic compounds. Organ cultures can bridge basic science with translational medicine and allow in vitro experiments while maintaining cells in their native tissue [39]. NP cultures confined in Dyneema jackets exhibit rather stable characteristics over sustained periods of time. Moreover, accessibility and low costs of bovine tissue, as well as the possibility to modulate the phenotype from early to advanced NP degeneration, make this model attractive. Although more research is needed, we conclude that these advanced NP cultures can be suitable for the efficacy screening of novel regenerative compounds in the NP tissue.

## 4. Materials and Methods

### 4.1. Bovine Nucleus Pulposus Tissue Culture

**Scheme 1:**
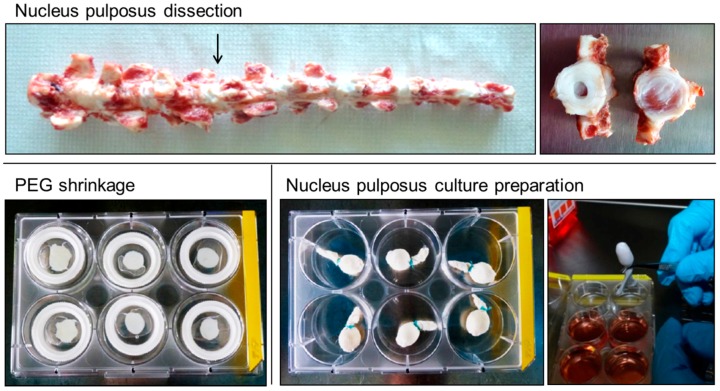
Preparation of bovine NP cultures: Bovine tails were dissected and NP tissue was isolated using an 8-mm biopsy punch. NP tissue was placed in 30% *w*/*v* polyethylene glycol (PEG) solution (100 min) to reduce water content. NPs packed in regenerated cellulose membranes and Dyneema jackets were sutured and placed in culture media.

NP cultures were prepared as previously described, with minor changes [49]. Bovine tails were obtained from the local slaughterhouse (18–24 month old cows), muscles and fat were removed and vertebrae were cut to obtain individual motion segments of the first 4–5 intact proximal discs. The motion segments were sterilized with betadine for 20 min and further washed under sterile conditions with 70% ethanol. Intervertebral discs were cut transversally, close to the endplate, and nucleus pulposus tissue was isolated using an 8-mm biopsy punch (8437995, Polymed, Anderson, SC, USA) and scalpel (Scheme 1A). NP biopsies were placed in 1.5 mL tubes and weighed.

For NP pre-shrinkage, dialysis membranes (Spectrapor 15 kDa MWCO/18 mm, 734-0507, VWR, Dietikon, Switzerland) were washed in PBS, opened with tweezers and one NP each was pushed inside using a custom-made cylindrical metal device. The Spectrapor membranes with NPs were then placed into membrane clips, closed and allowed to shrink in a 6-well plate with 5 mL of 30% *w*/*v* polyethylene glycol (PEG) solution in PBS for 100 min (Scheme 1B). After that, the membrane clips were opened, the membranes were cut open and the NP biopsies were weighed again. NP biopsies were placed on a regenerated cellulose membrane (97010-104 RC58, 0.2 µm pore size, GE Healthcare, Little Chalfont, UK) and folded into the membrane. Then NPs were inserted into the custom-made Dyneema jacket (UHMWPE fiber, Dyneema, DSM, Geleen, The Netherlands). The open end of the jacket was twisted once and turned inside-out again over the sample. This step was repeated once more to create three layers of the jacket, preventing excessive swelling. A sterile needle and thread was used to suture the jacket closed (Scheme 1C).

The encapsulated NP cultures were placed into 6-well plates with 10 mL of DMEM/F-12 (D8437, Sigma, St. Louis, MO, USA) supplemented with 10% fetal calf serum (FCS) (F7524, Sigma), penicillin (100 units/mL), streptomycin (100 μg/mL) and ampicillin (250 ng/mL, 15240-062, Gibco, Carlsbad, CA, USA). 2% FCS was used for experiments with IL-1β and TNF-α. NP tissues were cultured for up to 21 days. At each time point (Days 3, 7, 14, and 21), NP cultures were harvested for analysis, weighed, immediately frozen in liquid nitrogen and stored at −80 °C.

### 4.2. Induction of Nucleus Pulposus Degeneration

**Scheme 2:**
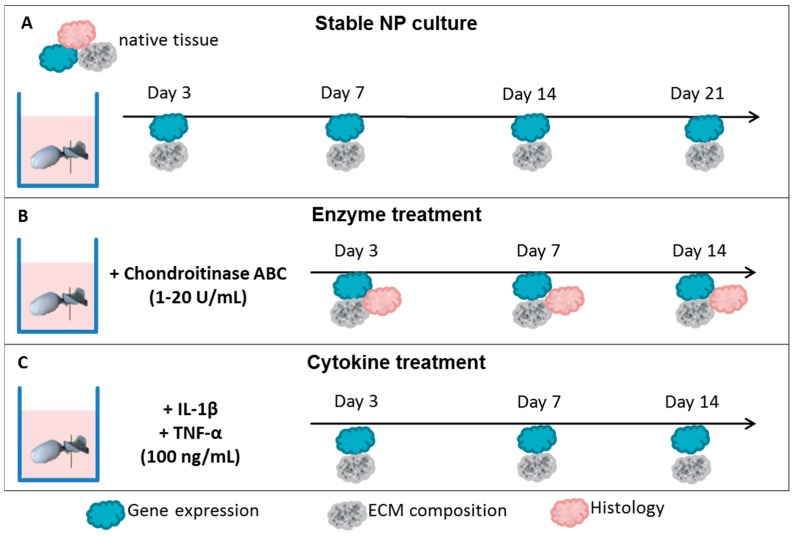
Experimental setup: (**A**) NP tissues were cultured up to 21 days to confirm the system’s stability; (**B**) Chondroitinase ABC was injected into NP cultures; and (**C**) TNF-α and IL-1β were added into the culture media. Gene expression and extracellular matrix (ECM) composition were analyzed on Days 0, 3, 7, 14, and/or 21. Histological analysis was performed to confirm GAG depletion in the enzyme treatment group. Epigallocatechin 3-gallate was used to counteract inflammation in cytokine treatment group.

To induce early degenerative changes of NP tissue, two approaches were tested: (1) enzyme treatment; and (2) cytokine treatment. In the first approach, NPs were injected with chondroitinase ABC (1–20 U/mL), which selectively removes chondroitin-4-sulfate, chondroitin-6-sulfate and dermatan sulfate, the major components of proteoglycans [67]. Chondroitinase ABC is a known inducer of disc degeneration in vitro and in vivo [67,68]. However, it is not known whether chondroitinase ABC can activate inflammatory responses in NP tissues. Lyophilized chondroitinase ABC from Proteus vulgaris (120 kDa, C3667, Sigma) was diluted according to the producer’s instructions in PBS and 1% BSA to 20 U/mL and stored as a stock solution at −20 °C until use. Twenty microliters of 1, 5, 10 or 20 U/mL chondroitinase ABC was injected with a 27 G needle in the center of the NPs before packing in Dyneema jackets. In the second approach, human recombinant TNF-α (17 kDa, 100 ng/mL, PHC3016, Gibco) and bovine recombinant IL-1β (31 kDa, 100 ng/mL, RBOIL1BI, Pierce, Woodland Hills, CA, US) were added to the culture media on Days 0 and 7. Re-swelling of the NP in the Dyneema jacket to its initial weight is thought to allow the media with IL-1β and TNF-α to actively enter the NP tissue. Samples were collected for analyses of degenerative responses and ECM composition on Days 0 (native), 3, 7, and 14. Epigallocatechin 3-gallate (EGCG, 10 µM), which inhibits inflammatory and catabolic responses in IVD cells in vitro [11], was selected to test the relevance and applicability of the degenerative NP culture model for therapeutic testing. The experimental setup is depicted in Scheme 2. The number of NP cultures used for each experimental group, treatment, and type of analysis is listed in Table 2.

### 4.3. RNA Isolation

RNA was extracted with the classical TRIzol/chloroform method, but optimized for bovine discs. Each bovine NP sample was quickly ground and pulverized in liquid nitrogen using custom-made grinders. The NP powder was transferred into 1 mL of TRIzol (15596018, Thermo Scientific, Waltham, MA, USA) and the mixture was further homogenized with a polytron 4 times/20 s (POLYTRON^®^ Kinematica, Luzern, Switzerland, PT 10/35 GT). During homogenization, samples were kept on ice. Homogenized samples were transferred to 15 mL tubes with 9 mL of fresh TRIzol, mixed and left at room temperature for 5 min. Then, 2 mL of chloroform (C7559, Sigma) was added, samples were vortexed and left 10 min on a shaker. After shaking, samples were centrifuged (4000× *g*/10 min) and the upper phase (5 mL) was transferred into new 15 mL tubes before adding 10 mL of 2-propanol (I9516, Sigma), followed by mixing, vortexing and centrifugation after 5 min at room temperature (4000× *g*/10 min). Isopropanol was discarded, 1 mL of 70% ethanol (51976, Sigma) was added, and samples were transferred into 2 mL tubes and centrifuged (7500× *g*/5 min). If the pellet contained white proteoglycans, samples were thoroughly vortexed for 2 min and a second TRIzol/chloroform isolation in smaller volume (2 mL tube) was performed for additional purification. Purified samples were centrifuged (7500× *g*/5 min), ethanol was aspirated and the pellets were dried for 5 min at room temperature. Dry pellets were then mixed with 30 µL of RNase-free water and immediately frozen at −80 °C until further analysis.

### 4.4. Gene Expression

As RNA yields were low, due to the low cellularity of the tissue and the high amount of proteoglycans [69], the whole RNA solution (30 µL) was reverse transcribed to cDNA using a reverse transcription kit (4374966, Applied Biosystems, Foster City, CA, USA). Resulting cDNA was diluted 3–5 times in RNase free water, mixed with primers and master mix (4352042, Applied Biosystems) and gene expression was measured by real-time PCR (CFX96 Touch™ Detection System, Biorad, Hercules, CA, USA). Bovine TaqMan primers for IL-6, IL-8, IL-1β, TIMP1, MMP1, MMP3, MMP13, ADAMTS4, PTGS2, iNOS, collagen 1, collagen 2 and aggrecan were employed to study the inflammatory and catabolic phenotype. S18, Actin and glyceraldehyde-3-phosphate dehydrogenase (GAPDH) were used as housekeeping genes (Table 3). Obtained C_q_ values were analyzed by the comparative method (2^−ΔΔCq^) and displayed as fold change to control or relative to IL-1β and TNF-α stimulation.

### 4.5. Extracellular Matrix Composition (Glycosaminoglycans, Hydroxyproline, DNA, and Water)

NP samples were dried at 60 °C overnight and weighed to determine dry weight. The dried samples were then digested in papain buffer (55 mM Na-Citrate, 150 mM NaCl, 5 mM EDTA, pH 6) with 5 mM l-cystein and 125 µg/mL papain from papaya latex (P-3125, Sigma) at 60 °C overnight. The digested samples were used to quantify the content of sulfated glycosaminoglycans (GAG), collagen (hydroxyproline) and DNA. GAGs were measured using dimethylmethylene blue (DMMB) assay with chondroitin sulfate as standard (C6737, Sigma) [70]. Samples were mixed with DMMB stock solution (10.5 mg DMMB, 2.5 mL absolute ethanol, 1.0 g sodium formate in 500 mL dH_2_O, pH 1.5) in a 96-well plate and the absorbance was measured at 595 nm. The collagen content was analyzed with the hydroxyproline (HYP) assay kit (MAK008, Sigma) and DNA was determined using the Quant-iT PicoGreen dsDNA assay kit (P11496, Thermo Fischer, Wayne, MI, USA) according to the manufacturer’s recommendations. The amounts of GAG, HYP, DNA and water were expressed per mg dry or wet weight of the tissue (for stable NP culture) or relative to the control (for degeneration groups) [49].

### 4.6. Histology

After harvesting, NPs were fixed in 4% buffered paraformaldehyde (48 h, 5 mL/sample). After fixation, specimens were washed, dehydrated and stored in 75% ethanol [71]. Before paraffin embedding, samples were cut transversally into two halves. After embedding, 3 µm sections were prepared with a microtome (CryoStar NX70, Thermo Scientific) and stained with Alcian blue/Picosirius red and Safranin O. Images were taken with a CCD camera mounted on an Olympus IX51 microscope (Volketswil, Switzerland).

### 4.7. Statistical Analysis

All data sets were tested for normal distribution with the Shapiro–Wilk test and found to be normally distributed. A Student’s *t*-test (2 groups) and a one-way ANOVA with Tukey post-hoc test (more than 2 groups) were performed to detect statistical significance of differences at *p* < 0.05.

## 5. Conclusions

Organ culture models are valuable tools for studying therapeutic strategies against degenerative disc disease. However, it is challenging to develop a cost-effective organ culture model that contains all main hallmarks of degenerative disc disease and is at the same time biologically relevant. In this study, an inflammatory NP culture model was established and validated with the anti-inflammatory compound EGCG. This NP culture model expresses inflammatory, catabolic, and pain-related genes that are involved in the progression of human degenerative disc disease. Composition and molecular diffusion distances of the bovine NP are comparable to the human NP. Moreover, this model is cost-effective and allows high throughput tests of molecular therapeutics. ECM anabolic agents and compound combinations can be tested in the NP cultures, in which TNF-α and IL-1β-induced inflammation will be combined with Chondroitinase ABC-induced loss of GAG.

## Figures and Tables

**Table 1:** Average cycle threshold values (Cq ± SD) for all tested and expressed genes in stable nucleus pulposus (NP) cultures (Day 3). GAPDH, glyceraldehyde 3-phosphate dehydrogenase; MMP3, matrix metalloproteinase 3; TIMP1, tissue inhibitor of matrix metalloproteinases.

Gene	Mean Cq ± SD	Gene	Mean Cq ± SD
*S18*	28.24 ± 5.22	*Collagen 2*	26.92 ± 2.85
*GAPDH*	29.69 ± 1.78	*Aggrecan*	27.13 ± 3.33
*Actin-β*	30.02 ± 2.98	*MMP3*	33.65 ± 3.55
*Collagen 1*	35.52 ± 3.14	*TIMP1*	27.78 ± 2.49

**Table 2:** Number of NP cultures used for each experimental group, treatment, and type of analysis. ECM = extracellular matrix; PBS, phosphate buffered saline; TNF-α, tumor necrosis factor alpha; IL-1β, interleukin-1 beta; EGCG, epigallocatechin 3-gallate.

Experimental Groups	Analyses	
**Treatments**	**Gene Expression**	**ECM Composition**
1. Native tissue 2. Stable NP culture	*n* = 8 Day 3: *n* = 8 Day 7: *n* = 8 Day 14: *n* = 8 Day 21: *n* = 8	*n* = 5 Day 3: *n* = 4 Day 7: *n* = 4 Day 14: *n* = 4 Day 21: *n* = 4
**Enzyme Treatment**		
1. PBS 2. Chondroitinase ABC 1 U/mL 3. Chondroitinase ABC 5 U/mL 4. Chondroitinase ABC 10 U/mL 5. Chondroitinase ABC 20 U/mL	Day 3: *n* = 4/treatment Day 7: *n* = 4/treatment Day 14: *n* = 4/treatment	Day 3: *n* = 7/treatment Day 7: *n* = 5/treatment Day 14: *n* = 5/treatment
**Cytokine Treatment**		
1. Control 2. TNF-α + IL-1β (100 ng/mL) 3. EGCG (10 µM) 4. TNF-α + IL-1β + EGCG	Day 3: *n* = 5/treatment Day 7: *n* = 5/treatment Day 14: *n* = 7/treatment	Day 3: *n* = 4/treatment Day 7: *n* = 4/treatment Day 14: *n* = 4/treatment
Total	*n* = 321 NPs	

**Table 3:** TaqMan primers and sequence numbers used for qPCR analysis.

Gene	Primer Seq. No.	Gene	Primer Seq. No.
*S18*	Bt03225193_m1	*ADAMTS4*	Bt03224693_m1
*GAPDH*	Bt03210919_g1	*MMP1*	Bt04259497_m1
*Actin-β*	Bt03279174_g1	*MMP3*	Bt04259495_m1
*Interleukin-6*	Bt03211904_m1	*MMP13*	Bt03214052_m1
*Interleukin-8*	Bt03211908_m1	*TIMP1*	Bt03223720_m1
*Interleukin-1β*	Bt03212741_m1	*Collagen 1*	Bt03214883_m1
*iNOS*	Bt03249584_m1	*Collagen 2*	Bt03251861_m1
*PTGS2*	Bt03214492_m1	*Aggrecan*	Bt03212186_m1

## Supplementary Materials

Supplementary materials can be found at www.mdpi.com/1422-0067/17/10/1460/s1.
