# Supplementary material for: An Inflammatory Nucleus Pulposus Tissue Culture Model to Test Molecular Regenerative Therapies: Validation with Epigallocatechin 3-Gallate
Source: Int J Mol Sci. 2016 Sep 27;17(10):1640. doi: 10.3390/ijms17101640 (PMC5085673; doi:10.3390/ijms17101640)
Supplement: Supplementary file 1 [file ijms-17-01640-s001.pdf]

Supplementary Materials: An Inflammatory Nucleus Pulposus Tissue Culture Model to Test Molecular Regenerative Therapies: Validation with Epigallocatechin 3-Gallate

Olga Krupkova, Marian Hlavna, Julie Amir Tahmasseb, Joel Zvick, Dominik Kunz, Keita Ito, Stephen J. Ferguson and Karin Wuertz-Kozak

Table S1. Average cycle threshold values (Cq ± SD) for all expressed genes in inflammation group. GAPDH, glyceraldehyde-3-phosphate dehydrogenase; MMP, matrix metalloproteinase; IL, interleukin; EGCG, epigallocatechin 3-gallate; TIMP1, tissue inhibitor of matrix metalloproteinases; PTGS2, prostaglandin-endoperoxide synthase 2; ADAMTS, a disintegrin and metalloproteinase with thrombospondin motifs; iNOS, inducible nitric oxide synthase.

Condition	Gene	Mean Cq ± SD	Condition	Gene	Mean Cq ± SD
Control	*S18*	27.49 ± 5.12	IL-1β	*S18*	27.61 ± 5.19
	GAPDH	27.74 ± 0.95		*GAPDH*	27.83 ± 1.17
	Collagen 1	43.46 ± 3.43		*Collagen 1*	43.38 ± 3.43
	Collagen 2	34.13 ± 3.29		*Collagen 2*	38.01 ± 3.55
	Aggrecan	36.14 ± 3.53		*Aggrecan*	35.59 ± 4.35
	MMP1	41.37 ± 8.87		*MMP1*	32.51 ± 12.11
	MMP3	43.61 ± 2.91		*MMP3*	32.47 ± 8.21
	MMP13	37.50 ± 10.18		*MMP13*	33.92 ± 5.44
	TIMP1	35.71 ± 9.91		*TIMP1*	31.41 ± 2.94
	IL-6	45 ± 0		*IL-6*	36.35 ± 4.49
	IL-8	45 ± 0		*IL-8*	38.76 ± 4.12
	PTGS2	43.13 ± 3.23		*PTGS2*	37.21 ± 2.21
	ADAMTS4	45 ± 0		*ADAMTS4*	43.57 ± 2.85
	iNOS	45 ± 0		*iNOS*	35.31 ± 1.54
EGCG	*S18*	27.69 ± 4.49	IL-1β + EGCG	*S18*	27.61 ± 5.25
	GAPDH	27.91 ± 0.45		*GAPDH*	27.84 ± 1.14
	Collagen 1	45 ± 0		*Collagen 1*	45 ± 0
	Collagen 2	36.62 ± 5.01		*Collagen 2*	37.86 ± 4.09
	Aggrecan	36.31 ± 4.43		*Aggrecan*	37.01 ± 3.71
	MMP1	39.28 ± 11.15		*MMP1*	37.95 ± 8.34
	MMP3	40.79 ± 5.05		*MMP3*	35.82 ± 7.13
	MMP13	37.84 ± 8.86		*MMP13*	35.23 ± 8.58
	TIMP1	36.31 ±7.25		*TIMP1*	32.83 ± 5.05
	IL-6	41.62 ± 4.65		*IL-6*	38.06 ± 3.95
	IL-8	44.41 ± 1.45		*IL-8*	42.25 ± 4.35
	PTGS2	41.67 ± 3.83		*PTGS2*	38.94 ± 2.61
	ADAMTS4	42.76 ± 4.46		*ADAMTS4*	43.33 ± 3.32
	iNOS	42.77 ± 4.44		*iNOS*	38.31 ± 4.57
